# Immunization with PfGBP130 generates antibodies that inhibit RBC invasion by *P. falciparum* parasites

**DOI:** 10.3389/fimmu.2024.1350560

**Published:** 2024-05-28

**Authors:** Yannick Johnson, Ahmad Rushdi Shakri, Sunthorn Pond-Tor, Anup Jnawali, Tanbir Najrana, Haiwei Wu, Jhasketan Badhai, Mohamad-Gabriel Alameh, Drew Weissman, Edward Kabyemela, Patrick Duffy, Michal Fried, Jonathan Kurtis, Dipak Kumar Raj

**Affiliations:** ^1^ Department of Pathology and Laboratory Medicine, Warren Alpert Medical School, Brown University, Providence, RI, United States; ^2^ Department of Internal Medicine, University of South Florida, Tampa, FL, United States; ^3^ Center for International Health Research, Rhode Island Hospital, Warren Alpert Medical School, Brown University, Providence, RI, United States; ^4^ Department of Medicine, University of Pennsylvania, Philadelphia, PA, United States; ^5^ Department of Pathology, Muhimbili University of Health and Allied Sciences, Dar es Salaam, Tanzania; ^6^ Laboratory of Malaria Immunology and Vaccinology, National Institute of Allergy and Infectious Diseases, National Institutes of Health, Rockville, MD, United States

**Keywords:** malaria, vaccine, blood stage malaria antigen, mRNA, growth inhibiting activity, *Plasmodium falciparum*

## Abstract

**Background:**

Despite decades of effort, *Plasmodium falciparum* malaria remains a leading killer of children. The absence of a highly effective vaccine and the emergence of parasites resistant to both diagnosis as well as treatment hamper effective public health interventions.

**Methods and results:**

To discover new vaccine candidates, we used our whole proteome differential screening method and identified PfGBP130 as a parasite protein uniquely recognized by antibodies from children who had developed resistance to *P. falciparum* infection but not from those who remained susceptible. We formulated PfGBP130 as lipid encapsulated mRNA, DNA plasmid, and recombinant protein-based immunogens and evaluated the efficacy of murine polyclonal anti-PfGBP130 antisera to inhibit parasite growth in vitro. Immunization of mice with PfGBP130-A (aa 111–374), the region identified in our differential screen, formulated as a DNA plasmid or lipid encapsulated mRNA, but not as a recombinant protein, induced antibodies that inhibited RBC invasion *in vitro*. mRNA encoding the full ectodomain of PfGBP130 (aa 89–824) also generated parasite growth-inhibitory antibodies.

**Conclusion:**

We are currently advancing PfGBP130-A formulated as a lipid-encapsulated mRNA for efficacy evaluation in non-human primates.

## Introduction

*Plasmodium falciparum* malaria remains a significant cause of morbidity and mortality in developing countries with over 620,000 deaths in sub-Saharan Africa in 2020 (1). Highly effective vaccines are urgently needed, yet the vaccine development pipeline is limited with most vaccine candidates in development targeting only four parasite antigens (2, 3). This situation mandates novel strategies to identify new candidate antigens.

Currently, the most advanced malaria vaccine, RTS,S, targets the circumsporozoite protein (CSP) expressed by sporozoites and generates antibodies that block sporozoite invasion of hepatocytes. While the potential role for cell-mediated immune responses has been sought, to date, these responses have not been reliably implicated in RTS,S-mediated protection (4). Critical challenges to targeting CSP include the following: 1) the short duration of exposure of CSP to circulating vaccine-induced antibodies (sporozoites invade hepatocytes within 20 min for IV injected and 120 min for ID-injected parasites (5)), thus requiring very high levels of specific antibody for vaccine efficacy (>100 EU/ml to prevent 50% of infections (6)), and 2) lack of expression of CSP on blood stage parasites, such that if a single sporozoite escapes vaccine-induced antibodies, an un-checked, blood stage infection can ensue. Notably, anti-CSP responses do not contribute to naturally acquired resistance, which is mediated by anti-blood-stage antibodies and provides broad protection to adults in holo-endemic areas. In the definitive Phase III trial of RTS,S, these challenges resulted in a vaccine efficacy of RTS,S against severe malaria in 17.3% of infants if given as four doses, which declined to only 10.3% if given as three doses—neither of these comparisons was statistically significant (7). Recently, vaccination with R21, a second formulation targeting the CSP protein, significantly reduced the incidence of clinical malaria (parasitemia with fever) by 74%–77% in a phase IIb trial conducted in a high, seasonal transmission setting (8, 9). Currently, the efficacy of R21 against severe malaria remains unknown.

Because parasites, which escape pre-erythrocytic vaccines can result in potentially fulminant blood-stage infections, there is an urgent need to develop blood-stage vaccines, which will attenuate clinical disease (10). Despite this need, the blood stage vaccine development pipeline is extremely limited, with only six actively recruiting clinical trials for *P. falciparum* blood-stage vaccines registered on clinicaltrials.gov. Of these active trials, five are targeting the Rh5 antigen, while one targets MSP-1. Both vaccine candidates are designed to attenuate parasite replication by generating humoral responses, which block merozoite invasion of erythrocytes. Previous studies have demonstrated significant antigenic variation in MSP-1 (11–13), and despite strong immunogenicity, this antigen failed to generate protection in Phase IIb studies (14). Rh5 has limited polymorphism in field isolates (15, 16), is essential for erythrocyte invasion (17), anti-Rh5 blocks erythrocyte invasion (18), vaccination with Rh5 protects against *P. falciparum* challenge in non-human primates (19), and vaccination of humans results in a modestly reduced *P. falciparum* replication rate in controlled human challenge studies, but only at high anti-Rh5 concentrations (20).

To identify new blood-stage vaccine candidate antigens, we applied our whole proteome differential screening method and identified PfGBP130 as a target of antibodies expressed by children who had acquired resistance to *P. falciparum* infection (21–23).

## Materials and methods

### Ethical approval

Protocols for the original longitudinal cohort study were approved by the International Clinical Studies Review Committee of the Division of Microbiology and Infectious Diseases at the US National Institutes of Health. Ethical clearance was obtained from the Institutional Review Boards of Seattle Biomedical Research Institute and the National Institute for Medical Research in Tanzania (Study No 1059357). Protocols for the use of animals were approved by the IACUC committee of Rhode Island Hospital (Study No 1758696).

### Study population

Subjects participated in the Mother–Offspring Malaria Studies (MOMS) project as described (24, 25).

### Inclusion criteria and clinical monitoring

We monitored N = 785 children for *P. falciparum* infection from birth up to 3.5 years of age as described (21). Briefly, blood smears were obtained every 2 weeks from birth to 1 year of age, and monthly thereafter. Routine blood samples were collected once every 6 months from 1.5 to 3.5 years of life.

### Blood collection and malaria assessment

Venous blood was collected every 6 months and stored at 4°C until processing as described (21).

*Selection of resistant and susceptible children:* The selection was performed based on blood films collected between the ages of 2 and 3.5 years as described (21).

### Library construction and differential screening

Phage display library construction and screening were performed as described (21). In our previous publication (21), we performed four rounds of positive selection followed by five rounds of negative selection, and this screen resulted in marked enrichment for high-affinity clones (44% of clones encoded PfGARP). To attenuate the strong enrichment observed after four rounds of positive selection and allow identification of phage clones with lower affinity or with slower growth characteristics, we sequenced phage that were isolated after three rounds of positive selection on plasma from resistant children and five rounds of negative selection on plasma from susceptible children.

### Expression and purification of recombinant PfGBP130-A

PfGBP130-A (aa 111–374) was codon optimized and cloned into the plasmid pJ411 (Atum) with N-terminal StrepTagII (8 aa) and C-terminal 10xHIS tags. Expression and purification were performed as described (21), except final purification was achieved by chromatography on a 5-ml Strep-TactinXT SuperFlow affinity column according to manufacturer’s instructions (IBA-Lifesciences). Purified recombinant protein, designated PfGBP130-A, was buffer exchanged into 10 mmol/L of sodium phosphate, 0.05% Tween 20, 3% sucrose, concentrated to 500 µg/ml using tangential flow ultrafiltration (filter area 50 cm (2), pore size 5 kDa, Pall), and lyophilized and stoppered under nitrogen.

### Expression and purification of recombinant PfGBP130-ecto and PfGARP-ecto

PfGBP130-ecto (aa 89–824) or PfGARP-ecto (aa 51–673) was codon optimized and cloned into the plasmid pD2610-v6 (Atum) with the N-terminal secretion signal from pHLsec (26) and a C-terminal 10xHIS tag. Endotoxin-free plasmid was transfected into HEK293 cells with lipofectamine (Invitrogen) according to manufacturer’s instructions. Culture supernatant was harvested on day 5 after transfection, and PfGBP130-ecto or PfGARP-ecto was purified by nickel affinity column chromatography. For PfGARP-ecto, we further purified the recombinant protein using hydrophobic and anion exchange chromatography as described (21). For both constructs, purified recombinant protein was buffer exchanged into 10 mmol/L of sodium phosphate, 0.05% Tween 20, 3% sucrose, concentrated to 500 µg/ml using tangential flow ultrafiltration (filter area 50 cm (2), pore size 5 kDa, Pall), and lyophilized and stoppered under nitrogen.

### Production of PfGBP130-A mRNA

mRNAs were produced as previously described (27) using T7 RNA polymerase (Megascript, Ambion) on a linearized plasmid encoding codon-optimized (28) PfGBP130-A or PfGBP130-ecto.

### Encapsulation of mRNA in LNPs

mRNAs were encapsulated in LNPs as previously described (21). mRNA–LNP formulations were stored at −80°C at a concentration of mRNA of ~1 μg/μl.

### Parasite strains and culture

*P. falciparum* strains (3D7, Dd2, D10, W2, and INDO) were obtained from MR4. Two parasite isolates (one from a child, NIH 04122821, and one from an adult, NIH 0710) were collected from our Tanzanian field site and culture adapted. The parasites were cultured *in vitro* according to the methods of Trager and Jensen with minor modifications (29). Briefly, parasites were maintained in RPMI 1640 medium containing 25 mmol/L of HEPES, 5% human O+ erythrocytes, 5% Albumax II (Invitrogen), 24 mmol/L of sodium bicarbonate, and 10 μg/ml of gentamycin at 37°C with 5% CO_2_, 1% O_2_, and 94% N_2_.

### Anti-PfGBP130 antisera

Mouse anti-PfGBP130-A antisera were produced by either DNA-, recombinant protein-, or mRNA- based immunization as described (21). For DNA immunization, we subcloned the open reading frame encoding PfGBP130-A (amino acids 111–374) into VR2001, transformed this into the host *Escherichia coli* NovaBlue (Novagen), and purified endotoxin-free plasmid (Endofree Giga, Qiagen).

All anti-sera were generated in BALB/cJ mice. For DNA immunization, mice were immunized at baseline with 100 μg of plasmid (25-μg intramuscular injection in each hind leg and 50-μg intradermal injection at the base of the tail) followed by 50-μg intradermal injections at the base of the tail every 2 weeks for a total of four doses. For protein immunization, recombinant PfGBP130-A was emulsified in an equal volume of TiterMax adjuvant (CytRx Corporation) and 50 μg was injected intraperitoneally at 2-week intervals for a total of three doses. For mRNA-based immunization, mice were immunized intradermally with 10 μg of lipid-encapsulated mRNA (see below) encoding PfGBP130-A (amino acids 111–374) or PfGBP130-ecto (amino acids 51–673) every 3 weeks for a total of three doses.

### Anti-PfGBP130 antibody assays

Bead-based anti-PfGBP130 antibody assays were performed according to our published methods (30) as described (21) using PfGBP130-A or PfGBP130-ecto as target antigens and PfGARP-ecto as a negative control protein.

### Growth inhibition assays

**Figure 1:**
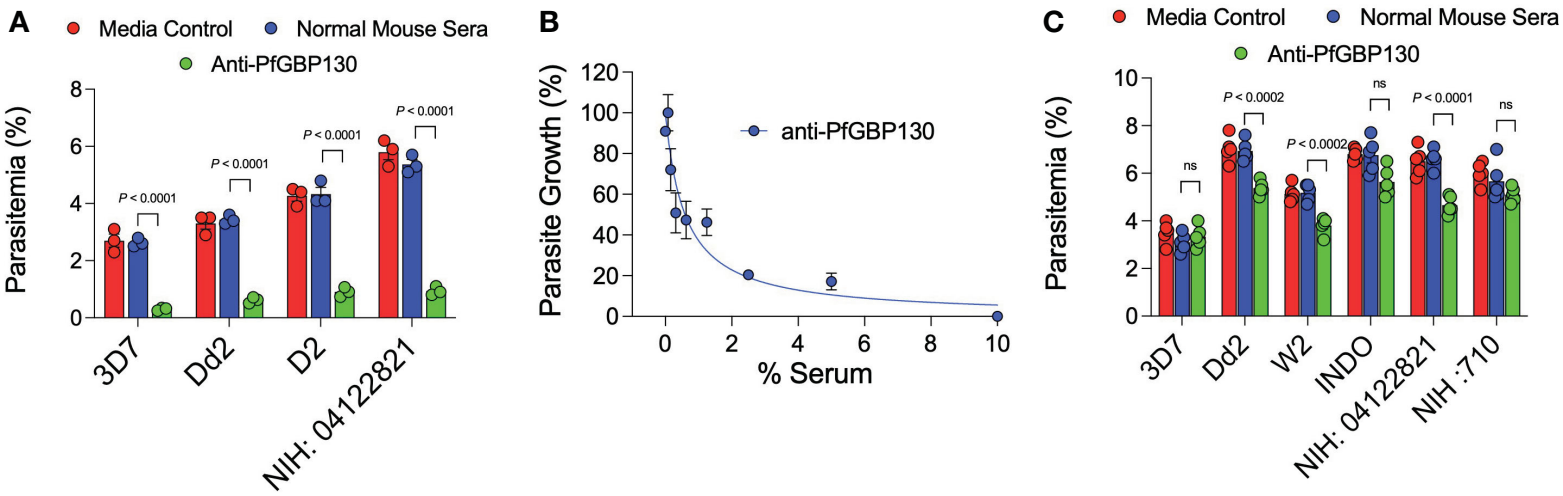
Anti-PfGBP130-A generated by DNA, but not recombinant protein immunization markedly attenuates parasite replication in multiple parasite strains. **(A)** Polyclonal anti-PfGBP130-A antibodies generated by DNA immunization in mice inhibit parasite growth by 79-89% in multiple parasite strains *in vitro*. Ring stage parasites at 0.3% parsitemia were cultured in the presence of anti-PfGBP130 mouse sera at 1:10 dilution. Negative controls included no anti-sera and normal mouse sera. **(B)** Parasite growth assay performed as in **(A)**, but with dilution series of anti-PfGBP130-A generated by DNA immunization. IC_50_ for inhibition of parasite growth was 0.6% serum. **(C)** Polyclonal anti-PfGBP130-A antibodies generated by recombinant protein immunization in mice resulted in no or modest (22-29%) growth inhibition in multiple parasite strains *in vitro*. For **(A, C)**, bars represent means, circles represent values from replicate wells, and error bars represent SEM. For **(B)**, circles represent means, error bars represent SEM. Results in **(A, C)** representative of 5 independent experiments.

Growth inhibition assays (GIA) were carried out with anti-PfGBP130 polyclonal serum generated by immunization with DNA-, recombinant protein-, or mRNA- based constructs (31–33) as described (21). Controls included no sera, normal mouse sera, sera generated by immunization with empty plasmid vector, and sera generated by immunization with LNPs containing mRNA encoding poly C. All sera were used at 10% final concentration, except as noted in the serial dilution experiment presented in **Figure 1B**.

### Merozoite invasion assays

To assess the ability of anti-PfGBP130 antisera to block merozoite invasion of erythrocytes, we incubated Percoll-/sorbitol-purified schizonts with fresh erythrocytes (final parasitemia 1%) in the presence of media control, normal mouse sera, or anti-PfGBP130-A sera generated by DNA immunization. Cultures were incubated for 12 h, blood films were prepared, stained with Giemsa, and newly invaded ring stage parasites were enumerated.

### Western blot

Parasite pellets were prepared using *in vitro* cultured 3D7 strain of *P. falciparum*. Parasite cultures at 10% parasitemia were treated with 0.15% saponin in PBS, pH 7.4 on ice for 10 min, followed by centrifugation (3,000*g*, 5 min). Parasite pellets were resuspended in cold PBS and centrifuged (3,000*g*, 5 min). Parasite pellets were dissolved in SDS sample-loading buffer with reducing agent (Bio-Rad), heated to 95°C for 10 min, and proteins were separated in 4%–12% gradient SDS–PAGE gels. Separated proteins were transferred to nitrocellulose membranes, and membranes were blocked in 5% BSA in 1X PBS (pH 7.4) and 0.05% Tween 20 for 1 h at 25°C. Membranes were probed with polyclonal anti-PfGBP130 generated by DNA-, mRNA- or recombinant protein-based immunization or normal mouse serum at 1:1,000 dilution in 1X PBS (pH 7.4), 0.05% Tween 20 overnight at 4°C. Rabbit polyclonal anti-actin diluted 1:3,000 was added as a loading control. Membranes were washed in 1X PBS (pH 7.4) and 0.05% Tween 20, and bound antibody was detected with anti-mouse IgG antibody conjugated to IRDye (1:3,000) and imaged on an LI-COR (Odyssey Imaging Systems).

### Immunofluorescence assays

Blood smears of asynchronous 3D7 strain parasite cultures were prepared, fixed in cold methanol for 15 min and probed with anti-PfGBP130-A generated by DNA, mRNA or recombinant protein-based immunization (dilutions tested from 1:50 to 1:200), and rabbit anti-PfMSP4 (obtained from MR4) diluted 1:500 in PBS, 5% BSA, pH 7.4. Blood smears were incubated with primary antibodies for 1 h at 25°C, washed three times in PBS, 0.05% Tween 20, and incubated with goat anti-mouse IgG conjugated with Alexa Fluor 488 (Molecular Probes) and goat anti-rabbit IgG conjugated with Alexa Fluor 594 (Molecular Probes). Blood smears were incubated for 10 min in 1 μg/ml of DAPI (Sigma-Aldrich) to label nuclei and cover slipped with ProLong Gold anti-fade reagent (Invitrogen). Blood smears were imaged using a confocal microscope (ZEISS LSM 900 Airyscan) equipped with a ×63 oil-immersion objective.

## Results

To identify novel vaccine candidates for *P. falciparum* infection, we pooled plasma collected from 2-year old children living in a holoendemic region of Tanzania who participated in a longitudinal cohort study (24). We pooled plasma from children who were highly resistant or highly susceptible to infection as assessed with monthly blood films from ages 2 to 3.5 years (**Supplementary Table S1**). Using these pooled plasmas, we performed differential biopanning using a *P. falciparum* blood-stage cDNA library constructed in the T7 bacteriophage using mRNA extracted from parasites freshly collected from our Tanzanian field site as described (21). Following three rounds of differential biopanning, we sequenced 100 clones (**Supplementary Table S2**) and identified PfGBP130 as a target of antibodies expressed by resistant, but not susceptible, children. After removal of ribosomal-related genes, PfGBP130 accounted for 10% of all clones sequenced.

**Figure 2:**
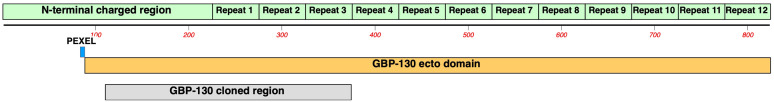
Domain structure of PfGBP130. PfGBP130 is an invariant, PEXEL containing merozoite surface antigen comprised of an N-terminal charged 225 aa domain followed by twelve copies of a 50 aa repeat domain. The PfGBP130 clone identified by differential screening (PfGBP130-A) encoded aa 111-374 which comprises the majority of the N terminal charged domain and three copies of the 50 aa repeat region.

PfGBP130 encodes an N-terminal PEXEL sequence (aa 84–88) followed by an ectodomain (PfGBP130-ecto, aa 89–824), which contains a charged 137-aa domain followed by 12 copies of a 50-aa repeat (**Figure 2**). The PfGBP130 clone we identified (PfGBP130-A) encoded aa 111–374, which comprises the majority of the N terminal charged domain and three copies of the 50-aa repeat region.

**Figure 3:**
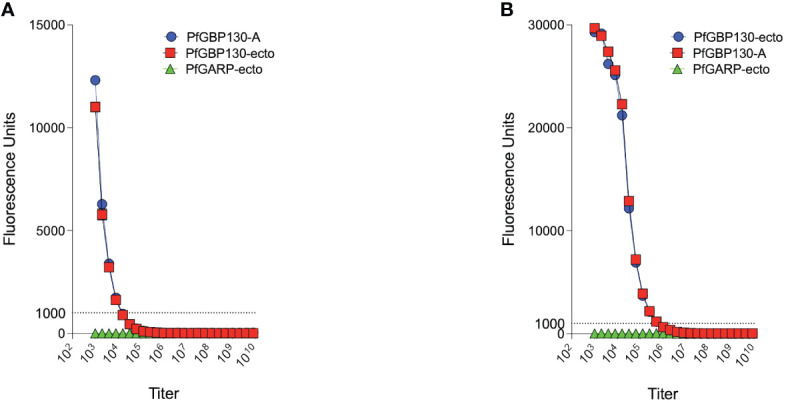
PfGBP130-A formulated as a DNA plasmid or recombinant protein is immunogenic in mice. **(A)** Mice immunized with a DNA plasmid encoding PfGBP130-A generated low titer (1:8,000) antibodies against PfGBP130-A and PfGBP130-ecto coated beads with no reactivity against negative control protein (PfGARP-ecto) coated beads. **(B)** Mice immunized with recombinant protein encoding PfGBP130-A generated high titer (1:512,000) antibodies against PfGBP130-A and PfGBP130-ecto coated beads with negligible binding to a negative control protein (PfGARP-ecto) coated beads.

Because of the proposed role for PfGBP130 in merozoite invasion of erythrocytes (34–39), we evaluated the ability of anti-PfGBP130 to attenuate parasite growth *in vitro*. We vaccinated mice with *E. coli*-expressed PfGBP130-A formulated in TiterMax® (CytRx Corporation) adjuvant or as a codon-optimized DNA vaccine construct. Both vaccination regimens generated antigen-specific antibody responses as measured by a bead-based antibody detection assay against PfGBP130-A, PfGBP130-ecto, or the negative control protein PfGARP-ecto (**Supplementary Figure S1**) with a titer of 1:8,000 for DNA and 1:512,000 for recombinant protein-based vaccination (**Figure 3**). In parasite growth inhibition assays, anti-PfGBP130-A antisera generated by DNA vaccination, significantly attenuated parasite growth by 79%–89% compared to controls, and this activity was consistent across multiple parasite strains including freshly isolated parasites (all *P* ≤ 0.0001, **Figure 1A**). This high degree of attenuation of parasite growth was confirmed using a second, independent lot of sera (titer = 1:64,000), which attenuated parasite growth by 95% (**Supplementary Figure S2**). Sera generated by immunization with empty DNA vector or DNA vector encoding an irrelevant *P. falciparum* gene (PfHISTc) had no impact on parasite growth (**Supplementary Figure S2**). In serial dilution experiments, the IC_50_ for parasite growth inhibition was 0.6% anti-PfGBP130-A sera, and at 10% serum, the parasite growth was inhibited by 100% (**Figure 1B**). In contrast, anti-PfGBP130-A antisera generated by recombinant protein-based immunization had no impact on parasite growth for three strains (3D7, INDO, and a freshly collected isolate, NIH 710) and had statistically significant, but very modest (22%–29%), attenuation of parasite growth for a further three strains (Dd2, W2, and a freshly collected field isolate, NIH 04122821, **Figure 1C**).

**Figure 4:**
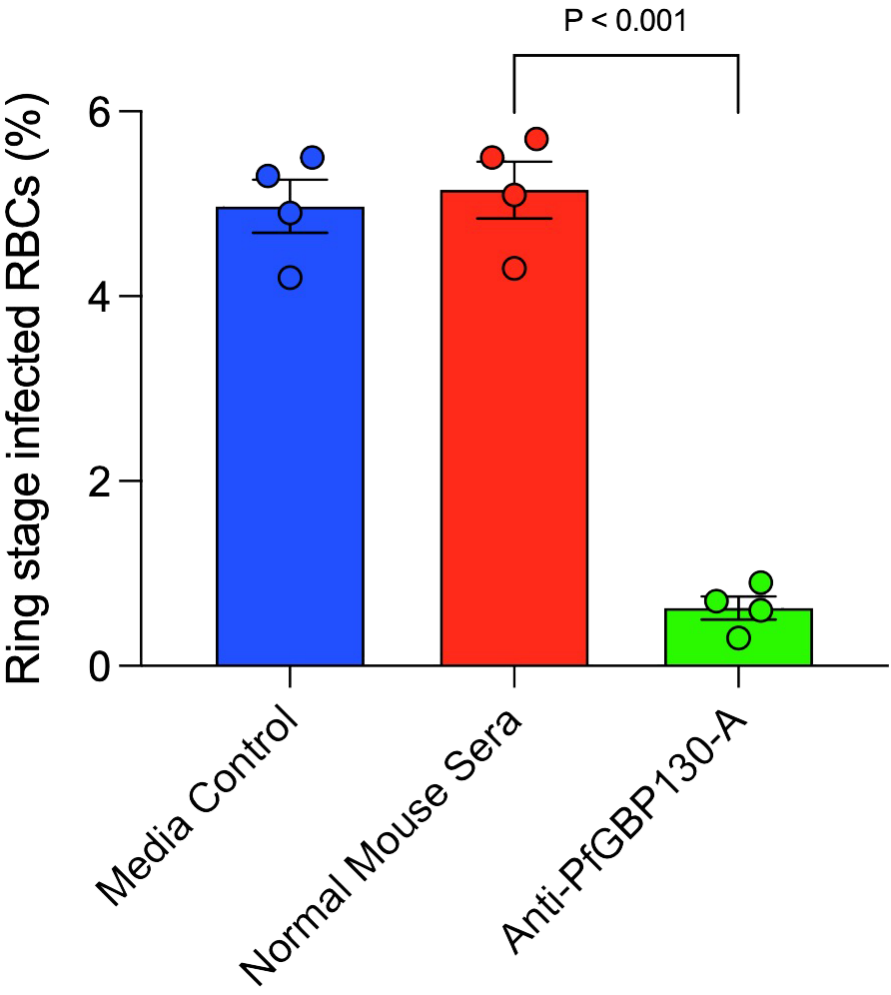
Antibodies to PfGBP130-A inhibit merozoite invasion. Polyclonal anti-PfGBP130-A antibodies generated by DNA immunization in mice inhibit merozoite invasion by 88% *in vitro*. Schizont stage parasites were cultured in the presence of anti-PfGBP130-A mouse sera at 1:10 dilution for 12 hours and newly invaded ring-stage parasites were enumerated. Negative controls included media alone and normal mouse sera. Bars represent means, circles represent values from replicate wells, and error bars represent SEM.

To assess whether anti-PfGBP130 blocked merozoite invasion of RBCs, we incubated Percoll-/sorbitol-purified schizonts with fresh RBC in the presence of media control, normal mouse sera, or anti-PfGBP130-A sera generated by DNA immunization. Parasite cultures were incubated for 12 h, and newly invaded ring-stage parasites were enumerated. Anti-PfGBP130-A inhibited merozoite invasion by *88%* compared to normal mouse sera (**Figure 4**).

**Figure 5:**
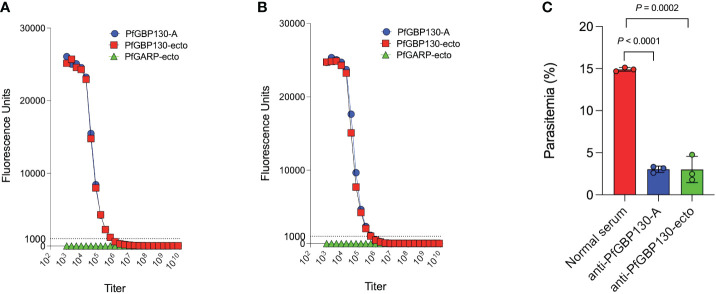
Immunization with PfGBP130-A or PfGBP130-ecto formulated as lipid encapsulated mRNA generates antibodies that markedly attenuate parasite replication. Immunization with **(A)** lipid encapsulated mRNA encoding PfGBP130-A or **(B)** PfGBP130-ecto generates high titer (both 1:512,000), specific antibodies against PfGBP130-A and PfGBP130-ecto coated beads with no reactivity against negative control protein (PfGARP-ecto) coated beads. **(C)** Polyclonal murine anti-PfGBP130 antibodies generated by immunization with LNPs containing mRNA encoding PfGBP130-A or PfGBP130-ecto inhibit parasite growth by 80% *in vitro*. Ring stage parasites were cultured in the presence of anti-PfGBP130 mouse sera at 1:10 dilution. Negative controls included no anti-sera and normal mouse sera. For **(A, B)** circles represent means, error bars represent SEM. For **(C)**, bars represent means, circles represent values from replicate wells, and error bars represent SEM. Results in **(C)** representative of 3 independent experiments.

Because PfGBP130 formulated as a recombinant protein did not generate antibodies with high growth inhibitory activity, we evaluated LNP-encapsulated mRNA based on our prior experience generating functional anti-malarial antibodies (21), as well as the recent success of mRNA as a delivery vehicle for SARS-CoV2 vaccines (40). We synthesized PfGBP130-A and PfGBP130-ecto as lipid-encapsulated mRNA formulations, and both constructs were highly immunogenic in mice, with both generating titers of 1:512,000 against PfGBP130-A- and PfGBP130-ecto-coated beads. Antibody titers following mRNA-based immunization were 8–64 times higher than titers generated by DNA immunization and were identical to titers following recombinant protein immunization (**Figures 5A, B**). In *in vitro* assays, polyclonal antibodies generated by both mRNA constructs inhibited parasite growth by 80% (**Figure 5C**), while LNP-encapsulated mRNA encoding poly C had no impact on parasite growth (**Supplementary Figure S2**).

To explore the discrepancy between antibody titer and parasite growth inhibition across the DNA-, mRNA-, and recombinant protein-generated anti-sera, we performed western blot analysis on extracts of schizont-stage 3D7 strain *P. falciparum* parasites. All three anti-PfGBP130-A antisera recognized a dominant protein migrating at 110 kDa in parasite-infected, but not uninfected, erythrocytes (**Supplementary Figure S3**). In immunofluorescence assays, none of the three anti-PfGBP130-A antisera generated a specific binding pattern when tested against infected erythrocytes (data not shown).

## Discussion

In the current report, we identified PfGBP130 as a target of antibodies expressed by children who had acquired resistance to, but not by children who remained susceptible to, *P. falciparum* infection.

Previously, we developed a whole proteome differential screening (WPDS) strategy, which identifies the subset of parasite antigens that are recognized by antibodies expressed by resistant individuals but not susceptible individuals (21–23). When employed with four rounds of positive selection, our WPDS method identified differentially recognized phage that were markedly oligoclonal with 44% of the clones encoding PfGARP (21). In the present study, to identify a broader clonal repertoire, including lower-affinity, slower-growing, and sub-dominant clones, we sequenced differentially recognized clones following only three rounds of positive selection. Using this approach, we identified PfGBP130 as a target of antibodies that block merozoite invasion resulting in arrested parasite replication.

PfGBP130 is an invariant merozoite surface antigen comprised of an N-terminal charged 225-aa domain followed by 12 copies of a 50-aa repeat domain (41). PfGBP130 is synthesized by trophozoite- and schizont-stage parasites and becomes associated with the surface of merozoites prior to egress (34). PfGBP130 interacts with the exofacial surface of erythrocytes (34) and appears to bind specifically to glycophorin A (35), though this interaction is disputed in some (36), but not other, reports (37). Rabbit polyclonal antibodies generated by immunization with an *E. coli*-expressed construct encoding 4.5 repeats of PfGPB130 inhibit merozoite invasion of erythrocytes (37), while a 19-amino acid synthetic peptides derived from the PfGBP130 repeat can also inhibit merozoite invasion (39).

Vaccination of non-human primates (*Aotus*) with a recombinant PfGBP130 polypeptide produced in *E. coli* that encoded three copies of the 50 amino acid repeat did not protect against challenge infection (42). These results suggest that potentially protective epitopes within PfGBP130 may not be contained in the repeat regions, but rather may be in the N-terminal**-**charged domain (aa 1–225), which overlaps with the region identified on our differential screen (aa 111–374).

In the current study, we evaluated the impact of three PfGBP130 vaccine delivery platforms on both immunogenicity as well as *in vitro* parasite growth arrest. Initially, we formulated PfGBP130-A as a codon-optimized DNA vaccine construct in a mammalian expression plasmid and as a recombinant protein expressed in *E. coli*. While immunization with recombinant protein generated higher antibody titers compared to DNA-based immunization (**Figure 3**), only antibodies generated by DNA-based vaccination significantly arrested parasite replication *in vitro* (**Supplementary Figure S2**, **Figure 1**), and this parasite growth arrest was mediated by blocking merozoite invasion of new erythrocytes (**Figure 4**). While the discordant results between DNA and recombinant protein immunization may have resulted from a misfolded *E. coli*-expressed protein, we have been unable to verify this assertion because we have not been successful in expressing PfGBP130-A in mammalian expression systems. Therefore, it remains unclear whether mammalian expression and/or the N-terminal region is required for generating antibodies that inhibit parasite growth.

To formulate PfGBP130 on a deployable and scalable delivery vehicle, we constructed lipid nanoparticle-encapsulated mRNA encoding PfGBP130-A (aa 111–374) or PfGBP130-ecto (aa 89–824). Both PfGBP130-A and PfGBP130-ecto, delivered as LNP-encapsulated mRNA-based vaccines, were highly immunogenic in mice (**Figures 5A, B**) and generated antibodies that significantly inhibited parasite replication *in vitro* (**Figure 5C**).

We did not find concordance between anti-PfGBP130-A antibody levels and *in vitro* parasite growth inhibition across the three delivery platforms evaluated. DNA-based immunization generated low-titer antibody levels, but these antibodies demonstrated high-parasite growth inhibition. mRNA-based immunization generated both high-titer antibody levels and high-parasite growth inhibition while protein-based immunization generated high titer antibody levels, but little to no parasite growth inhibition. The reactivity of anti-sera to native PfGBP130 in parasite extracts was similar in both specificity and intensity across the three delivery platforms (**Supplementary Figure S3**), and none of the antisera reacted with parasites in immunofluorescence assays. We are currently conducting linear epitope-mapping studies to further explore the discordance between anti-PfGBP130 antibody level and functional activity across the three delivery platforms.

The COVID-19 pandemic brought the benefits of lipid-encapsulated mRNA as a vaccine delivery platform into sharp focus. These benefits include rapid production of GMP-grade vaccine, remarkably high antibody titers following one or two doses, and the ability to easily alter the delivery payload with new variant sequences. The vaccine mobilization effort for COVID-19 also addressed several significant hurdles for mRNA-based vaccines, including the lack of global GMP production facilities, the absence of regulatory approval pathways, and the absence of significant safety data in large-scale trials in humans.

The current malaria vaccine landscape includes two approved, modestly effective pre-erythrocytic vaccines (RTS,S and R21). Because pre-erythrocytic vaccines do not confer sterile protection, there is an urgent need to address the blood-stage infections which will develop from parasites that escape these vaccines and infect hepatocytes with the goal of incorporating effective blood stage components into current pre-erythrocytic vaccines.

Based on our data demonstrating anti-PfGBP130-A antibodies generated by lipid-encapsulated mRNA-based vaccination attenuate parasite growth, we are now advancing this antigen/delivery platform combination toward vaccine trials in non-human primates.

## Data availability statement

The original contributions presented in the study are included in the article/**Supplementary Material**, further inquiries can be directed to the corresponding author/s.

## Ethics statement

The studies involving humans were approved by The International Clinical Studies Review Committee of the Division of Microbiology and Infectious Diseases at the US National Institutes of Health, and ethical clearance was obtained from the Institutional Review Boards of Seattle Biomedical Research Institute and the National Institute for Medical Research in Tanzania. The studies were conducted in accordance with the local legislation and institutional requirements. Written informed consent for participation in this study was provided by the participants’ legal guardians/next of kin. The animal study was approved by the IACUC committee of Rhode Island Hospital. The study was conducted in accordance with the local legislation and institutional requirements.

## Author contributions

JK: Conceptualization, Formal analysis, Funding acquisition, Methodology, Project administration, Resources, Supervision, Validation, Visualization, Writing – original draft. YJ: Methodology, Visualization, Writing – review & editing. AS: Methodology, Writing – review & editing. SP-T: Methodology, Writing – review & editing. AJ: Methodology, Writing – review & editing. TN: Methodology, Validation, Writing – review & editing. HW: Methodology, Supervision, Writing – review & editing. JB: Investigation, Methodology, Visualization, Writing - review & editing.. M-GA: Methodology, Writing – review & editing. DW: Methodology, Writing – review & editing. EK: Formal analysis, Methodology, Writing – review & editing. PD: Conceptualization, Data curation, Funding acquisition, Methodology, Project administration, Resources, Supervision, Writing – review & editing. MF: Conceptualization, Data curation, Investigation, Methodology, Project administration, Supervision, Writing – review & editing. DR: Conceptualization, Funding acquisition, Methodology, Writing – review & editing.
