# Supplementary figures and images for: Immunization with PfGBP130 generates antibodies that inhibit RBC invasion by P. falciparum parasites
Source: Front Immunol. 2024 May 28;15:1350560. doi: 10.3389/fimmu.2024.1350560 (PMC11165087; doi:10.3389/fimmu.2024.1350560)

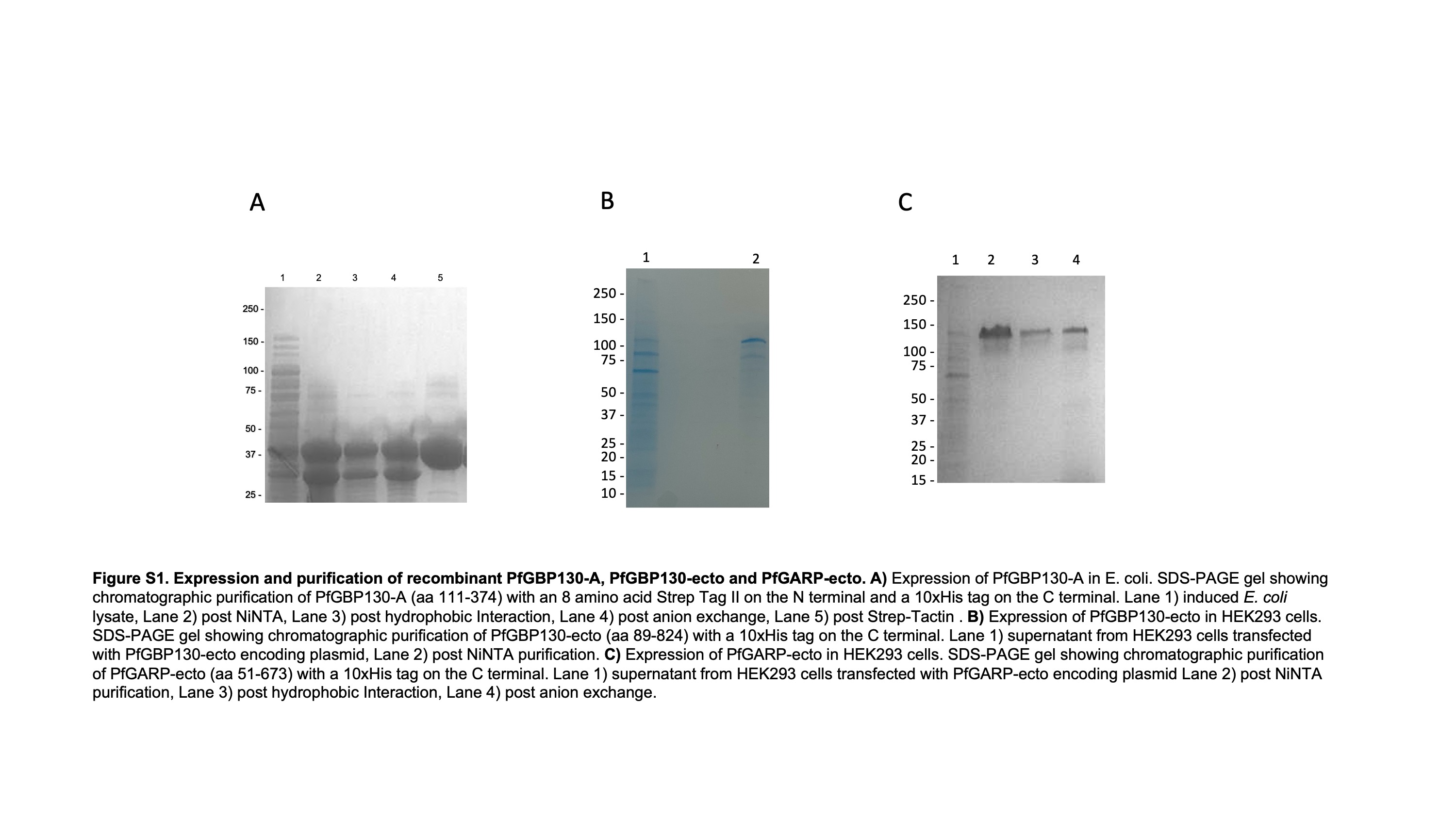

Supplement: Supplementary file 1 [file Image_1.jpeg]

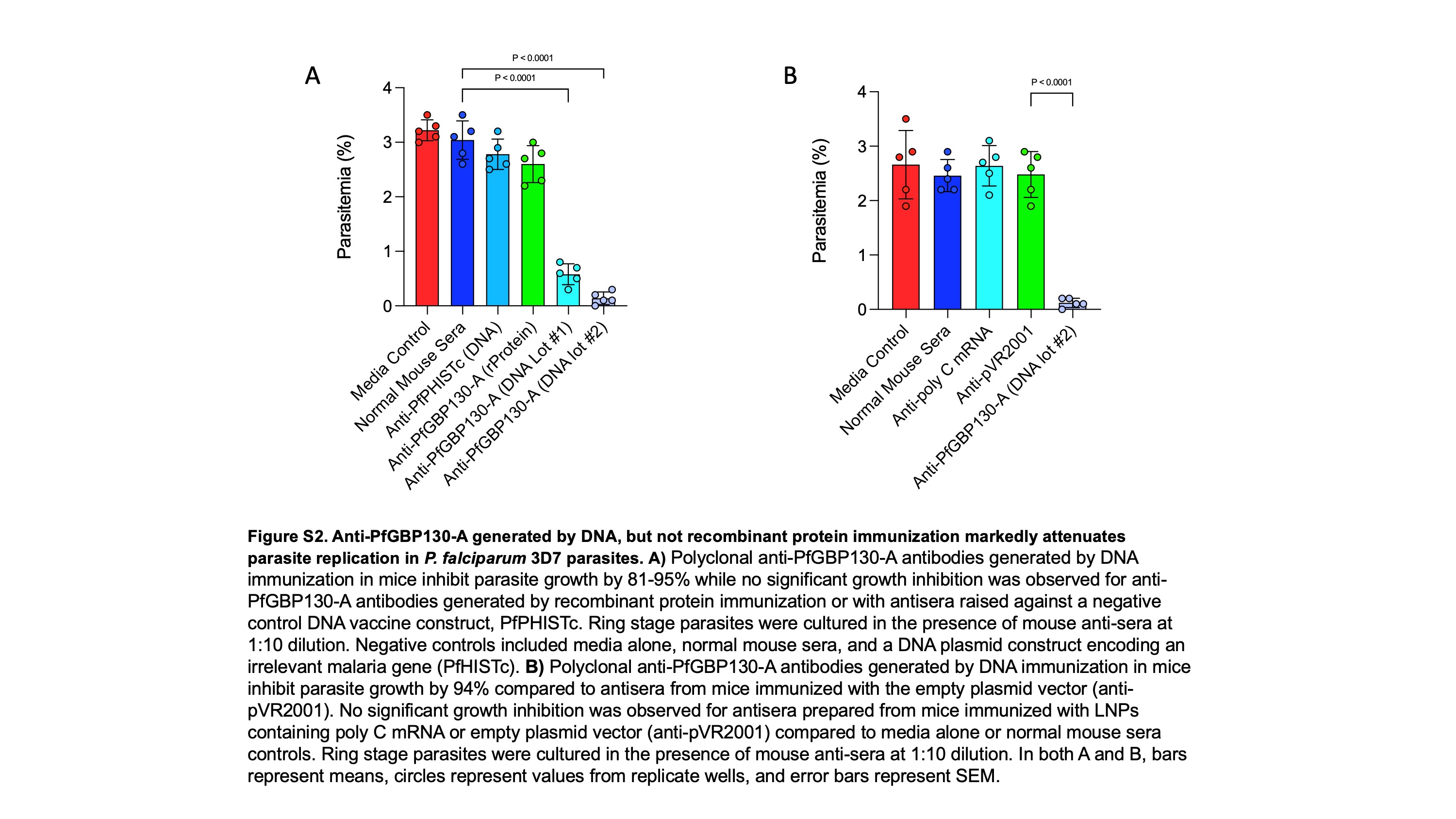

Supplement: Supplementary file 2 [file Image_2.jpeg]

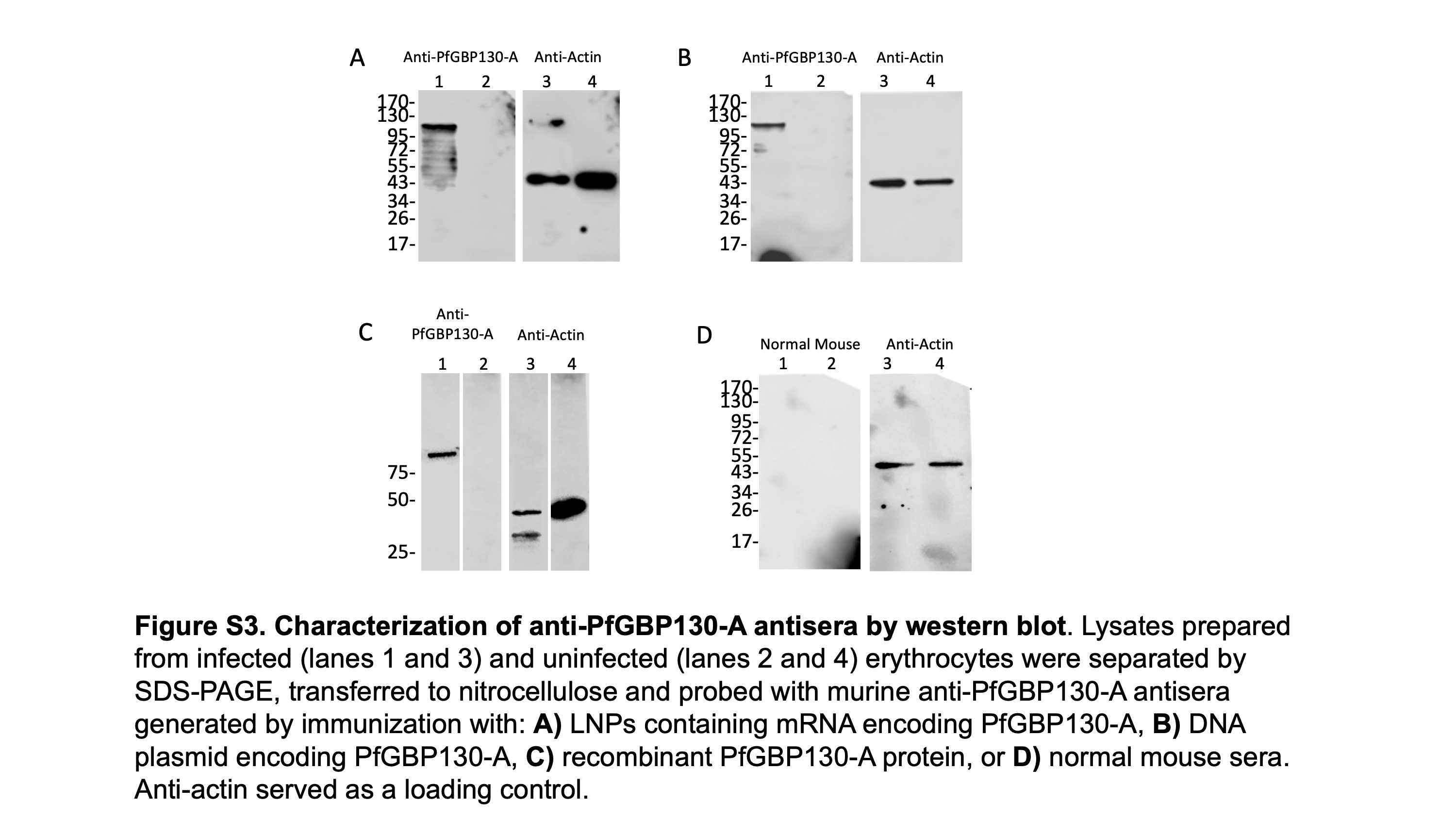

Supplement: Supplementary file 3 [file Image_3.jpeg]

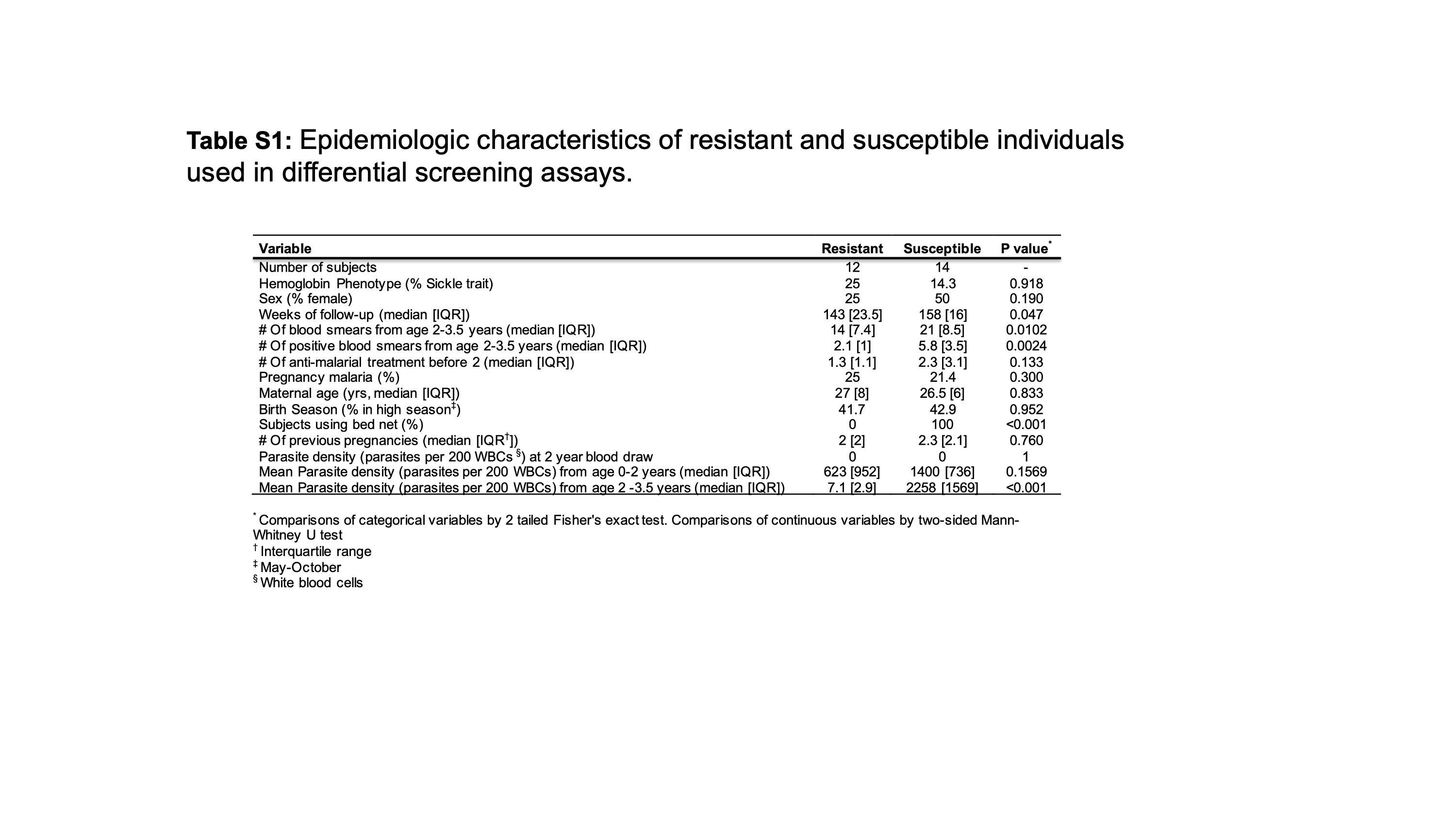

Supplement: Supplementary file 4 [file Image_4.jpeg]

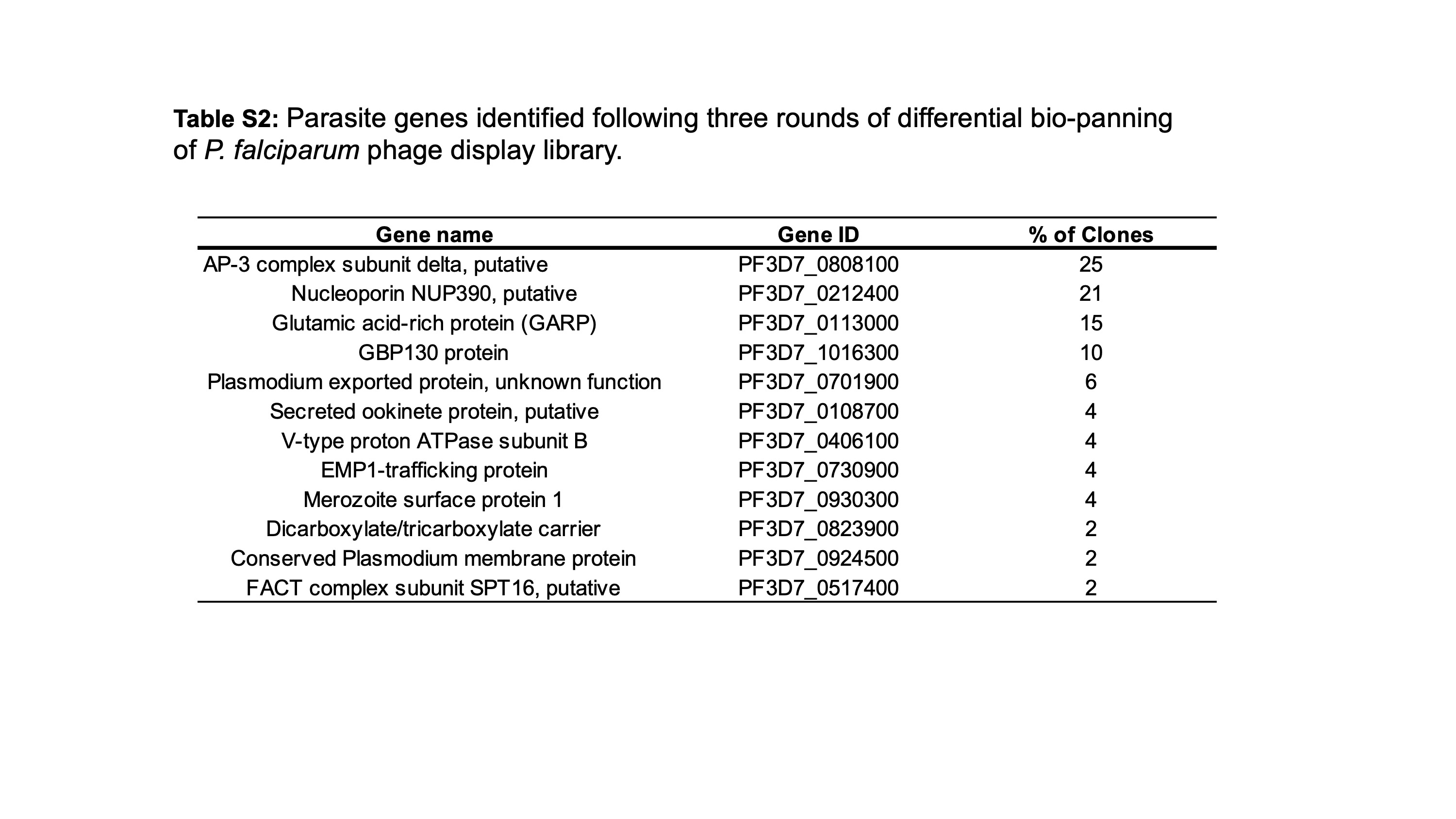

Supplement: Supplementary file 5 [file Image_5.jpeg]
